# Predictive habitat suitability models to aid conservation of elasmobranch diversity in the central Mediterranean Sea

**DOI:** 10.1038/srep13245

**Published:** 2015-08-14

**Authors:** V. Lauria, M. Gristina, M. J. Attrill, F. Fiorentino, G. Garofalo

**Affiliations:** 1 Institute for Coastal Marine Environment (IAMC), National Research Council (CNR), Via L. Vaccara n 61, Mazara del Vallo (TP), 91026, Italy; 2 Marine Institute, Plymouth University, Level 3, Marine Building, Drake Circus, Plymouth, PL4 8AA.

## Abstract

Commercial fisheries have dramatically impacted elasmobranch populations worldwide. With high capture and bycatch rates, the abundance of many species is rapidly declining and around a quarter of the world’s sharks and rays are threatened with extinction. At a regional scale this negative trend has also been evidenced in the central Mediterranean Sea, where bottom-trawl fisheries have affected the biomass of certain rays (e.g. *Raja clavata*) and sharks (e.g. *Mustelus* spp.). Detailed knowledge of elasmobranch habitat requirements is essential for biodiversity conservation and fisheries management, but this is often hampered by a poor understanding of their spatial ecology. Habitat suitability models were used to investigate the habitat preference of nine elasmobranch species and their overall diversity (number of species) in relation to five environmental predictors (i.e. depth, sea surface temperature, surface salinity, slope and rugosity) in the central Mediterranean Sea. Results showed that depth, seafloor morphology and sea surface temperature were the main drivers for elasmobranch habitat suitability. Predictive distribution maps revealed different species-specific patterns of suitable habitat while high assemblage diversity was predicted in deeper offshore waters (400–800 m depth). This study helps to identify priority conservation areas and diversity hot-spots for rare and endangered elasmobranchs in the Mediterranean Sea.

Commercial fisheries have dramatically impacted elasmobranch populations worldwide; with high capture and bycatch rates, the abundance of several species is rapidly declining^1^,^2^. Moreover, elasmobranchs are more vulnerable to overfishing than many teleost fish species owing to their specific biological and life-history characteristics (i.e. slow population growth rate, late age at maturity, longevity, low fecundity and long gestation period^3^,^4^). Most elasmobranchs are upper-level predators, so their removal from the marine food web can induce changes at an ecosystem level (i.e. affect the dynamics of their prey^3^ as well as lead to loss of biodiversity and productivity of marine systems^5^). Commercial fisheries directly impact elasmobranch populations by removing large individuals and, indirectly, as they form a large portion of the bycatch of other demersal fisheries^6^. Globally there is a major concern about the status of elasmobranch stocks in response to fisheries impact^7^. This is due to the fact that the abundance of several species is rapidly declining and about a quarter of the world’s sharks and rays are threatened with extinction^8^,^9^. At a regional scale this negative trend has also been evidenced in some areas of the Mediterranean Sea^2^,^10^,^11^, where mixed fisheries have critically impacted sharks and rays causing the disappearance of certain sharks species (e.g. *Mustelus mustelus*)^12^, as well as the overexploitation of some ray species (e.g. *Raja clavata*)^12^,^13^.

The Mediterranean Sea is an important area for elasmobranchs as it supports a large community of about 86 species^12^; however, according the International Union for Conservation of Nature (IUCN) Red List assessments, 46% of sharks and related species are classified as “Critically Endangered”, “Endangered” or “Vulnerable”. Furthermore for some elasmobranch species, the information available is very poor and 30% of these have been defined as “Data Deficient” (lack of sufficient information)^14^. The need to put into place effective long-term conservation plans for sharks, rays and chimaera populations was highlighted by the European Commission in 2009, with the first Action Plan for the conservation and management of elasmobranchs^15^. This framework aims to restore elasmobranch stocks under threat and set guidelines for the sustainable management of concerned fisheries in European waters. For these reasons, identifying and mapping elasmobranch sensitive habitats (i.e. areas essential to the ecological and biological requirements of at least one of the life stages and/or important for the recovery and long term sustainability of the species) in the Mediterranean can aid marine resource conservation as well as helping to improve our understanding of their relationship with the marine environment^14^.

Species ecological niche (defined as set of conditions necessary for a species survival and reproduction^16^) is the result of the combined action of external factors (e.g. environmental conditions or food availability^17^) and internal factors (e.g. population size, density dependent effects^18^). Habitat suitability models are widely used in both terrestrial and marine systems to quantify a species realised niche (sensu Hutchinson^19^), species-environment relationships and predicting species occurrence and/or density at unsurveyed locations^20^,^21^,^22^. The application of such models allows to characterise species geographical patterns, to identify spatial ontogenetic shifts of commercially exploited fish species^23^ and to test the effect of climate change on species distribution^24^. Moreover, habitat suitability models have the potential to become an essential tool to support management decisions and conservation measures in the framework of marine spatial planning^25^.

Information exists on the distribution patterns of a few elasmobranch species for some areas of the Mediterranean, but this is limited to the most frequently caught species (e.g. *Scyliorhinus canicula*, *Raja asterias*, *Galeus melastomus*; *Etmopterus spinax*^26^,^27^,^28^;) and not much is known on the habitat requirements of rare or endangered species^29^ (e.g. *Squalus blainvillei, Centrophorus granulosus*). In this study, long-term data (19 years) of a fishery-independent bottom trawl survey were used to predict the preferential habitat (the portion of potential habitat used on average over time) of nine elasmobranch species (1 chimaera, 4 rays and 4 sharks) as a function of five environmental variables (i.e. depth, sea surface temperature, surface salinity, slope and rugosity) in the central Mediterranean Sea. In addition, the habitat suitability of whole elasmobranchs community (assemblage diversity) (37 species; Table 1) was also modelled and mapped for the area.

This study helps to identify some of the distribution hot-spot for elasmobranchs in the central Mediterranean and provides a modelling framework for conducting similar studies. Moreover, it adds important knowledge on the species-environment relationship of some of elasmobranch species which are poorly understood (i.e. defined as data deficient from IUCN) which can support future conservation plans under the Marine Strategy Framework Directive^30^.

## Material and Methods

### Study area

**Figure 1:**
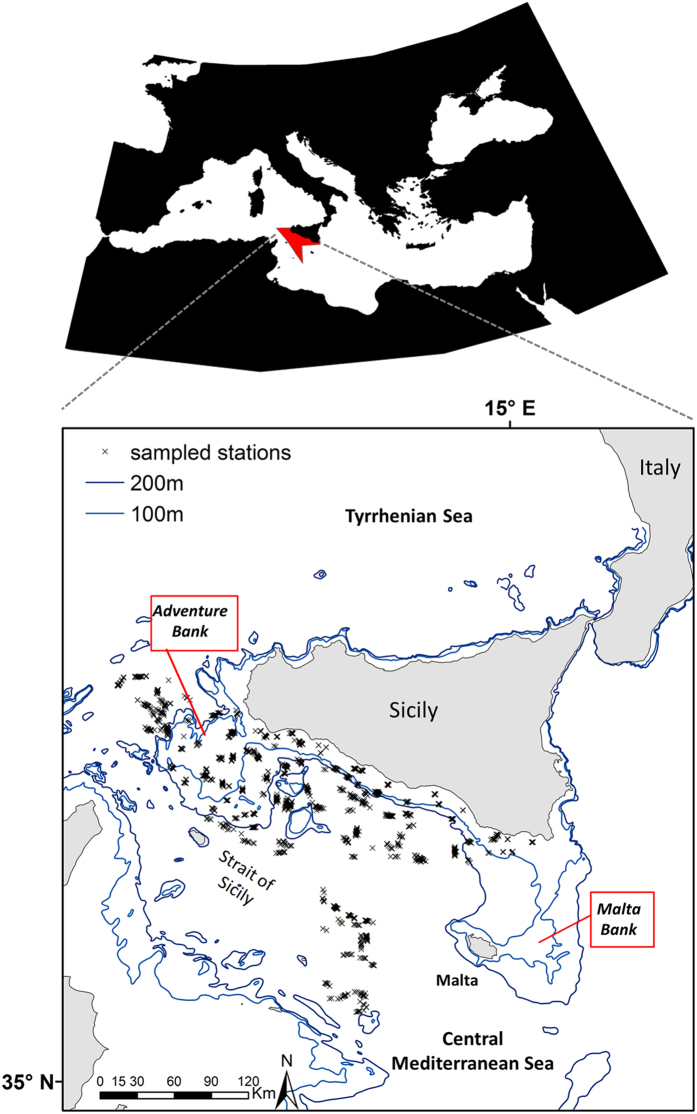
Location of the study region within the Strait of Sicily (Central Mediterranean Sea). This area corresponds to the Geographic Sub Area (GSA) 16 and extends for about 34,000 km^2^. Trawl stations sampled during the MEDITS Survey (1994–2011) are also showed with x. This map was created with ArcGIS version 10.2.2 by Valentina Lauria.

The study area is located in the central Mediterranean Sea and comprises the northern side of the Strait of Sicily between 34°59′–38°00′ N and 10°59′–15°18′W (Fig. 1). This area corresponds to the Geographic Sub Area (GSA) 16 of the General Fisheries Commission for the Mediterranean^31^ and extends for about 34,000 km^2^. It is characterised by complex seafloor morphology and hydrodynamic processes^32^, with a wide range of depths including a shallow bank in the western part (about 100 m depth named Adventure Bank) and deeper areas in the southeast (about 1800 m; Fig. 1). The Strait of Sicily is a particularly important area for biodiversity: it supports rich and diversified assemblages of fish, crustaceans and cephalopods^33^,^34^,^35^, as well as a large elasmobranch community^27^ (Table 1) many of which are listed in IUCN http://www.iucnredlist.org as “Data deficient”, “Near Threatened” and “Vulnerable”. Since the early eighties this area has been intensively exploited by many demersal fisheries (mainly bottom trawl)^36^,^37^, including the “Mazara del Vallo”, one of the largest and most active fleets in the Mediterranean^38^.

### Survey data

Since 1994 the area has been investigated under the Mediterranean International Trawl Survey program (MEDITS^39^). This survey is carried out annually in late spring-early summer, see “Supplementary information” (Table S1 online), and takes place in several areas of the Mediterranean Sea using a standardised sampling methodology^40^. It provides a long-term dataset of fishery-independent indices relating to demersal species abundance, demographic structure and spatial distribution. In GSA16, sampling stations are replicated each year according to a stratified random sampling design based on five depth strata: 10–50 m, 51–100 m, 101–200 m, 200–500 m, 500–800 m, where the number of hauls is proportional to the area of each stratum (Fig. 1). A total of 55–120 stations (haul duration = 30–60 min hauls; trawl speed = 5.6 kmh^−1^) was sampled each year (Fig. 1) on board the commercial stern trawler Sant’Anna. The gear was a bottom trawl with a high (2.5–3 m) vertical opening and 20 mm side diamond stretched mesh in the cod-end. At each trawl station, fish species were sorted, weighed, counted and measured. Elasmobranch densities, or relative abundance, from a total of 1345 trawl hauls covering the period 1994 to 2011 was expressed as numbers of individuals per km^2^ (Nkm^−2^). A total of 37 elasmobranch species were caught during the MEDITS survey (Table 1) with percentage occurrence (described as the number of hauls in which the species was found) varying between 0.07–43.57% (Table 1). To construct the habitat models only species with percentage of occurrence >5% and defined as “Near Threatened”, “Vulnerable”, “Critically Endangered” or “Data Deficient” in the IUCN Red Lists were selected (Table 2).

### Community diversity

Species richness (S) was computed for the elasmobranch community (37 species; Table 1) at each sampling station. This index describes the community in terms of the actual number of species included in any particular sample and was used to predict the habitat suitability of the whole elasmobranchs community.

### Environmental predictors

**Figure 2:**
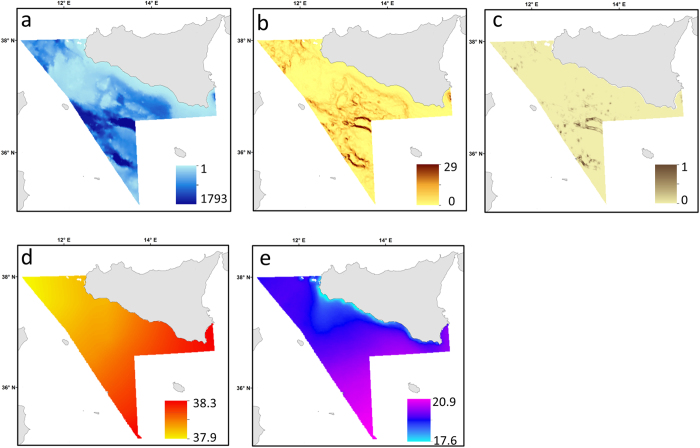
The spatial patterns of the environmental variables used to map the habitat models. These include (**a**) depth (m); (**b**) slope (degrees) values range from to 0° to 90° with low slope values corresponding to flat terrain while higher to steeper terrain); (**c**) Rugosity values range from 0 (no terrain variation) to 1 (complete terrain variation); (**d**) Salinity (PSU); (**e**) Satellite derived sea surface temperature (°C). These maps were created with ArcGIS version 10.2.2 by Valentina Lauria.

For habitat modelling, depth, slope, rugosity, sea surface salinity and Sea Surface Temperature (SST) were used as predictors of elasmobranch habitat suitability (Fig. 2). Given the limited geographical extent of the study area (about 3° of latitude and 4° of longitude), biogeographic gradients in species distribution patterns were considered not observable and the effects of latitude or longitude irrelevant with respect to other more local variables^41^ (e.g. depth, rugosity); hence geographical coordinates were not used as predictive variables. ArcGIS’s implementation of the Albers Equal Area Conic projection (ED50) was chosen as appropriate for use within the regional extent of our study. This is an equal-area map projection and uses two standard parallels designed to minimise area distortions at mid-latitudes with east-west orientation. Digital continuous maps of depth were derived from a re-projection of the MARSPEC database, available at http://www.marspec.org/. MARSPEC is a world ocean dataset with a spatial resolution of 30 arc-second developed for marine spatial ecology^42^. Extracted raster size for estimation of benthic variables was 866 × 866 m. Depth (Fig. 2a) is one of the main environmental gradients which controls species distribution and it has been identified as key factor to determine elasmobranchs spatial patterns^28^, with bigger/older individuals preferring deeper waters^43^. In this study we define three depth intervals: shallow waters (0–200 m), medium depth waters (201–600 m) and deeper waters (>600 m).

Bathymetry derived parameters (e.g. slope, rugosity) are indicative of seabed morphology and have been used as predictors of fish species distribution and suitable habitat^26^,^41^,^44^,^45^,^46^. Slope and rugosity (Fig. 2b,c) were derived from the continuous depth map using the Benthic Terrain Modeller tool in ArcGIS 10.1. Slope (expressed in degrees with values from 0° to 90°) describes the rate of change in elevation over distance. Low values of slope correspond to flat ocean bottom (or areas of sediment deposition) while higher values indicate potential rocky ledges. Rugosity (defined as the ratio between surface and plan area of square cells) provides an indicator of the bumpiness and complexity of the seafloor and emphasizes small variations in the seabed terrain. Rugosity values range from 0 (no terrain variation) to 1 (complete terrain variation), with typical values for natural terrains ranging between 0 and about 0.4 (Fig. 2c). Generally, soft seabed substrata correspond to low terrain rugosity and potential rocky seabed to high terrain rugosity. This parameter has been used as predictor of species distribution^44^,^47^,^48^ and is considered to have a strong utility as abiotic surrogate of benthic biodiversity when detailed information on sediment type is not available^41^,^45^,^46^.

Salinity and SST are strongly related to marine system productivity as they can affect nutrient availability, metabolic rates and water stratification^49^,^50^. These environmental factors have been shown to have an effect on demersal species distribution patterns^23^,^51^ as well as elasmobranch habitat suitability^26^,^52^. Annual maps of salinity (expressed in Practical Salinity Unit) were constructed by averaging monthly continuous digital maps (downloaded from the website http://iridl.ldeo.columbia.edu). SST maps (in °C) for each year were derived from the German Aereospace Agency (DLR) satellite data archive EOWEB available at http://eoweb.dlr.de:8080/index.html. For each year (1994–2011) values of SST and salinity were extracted in ArcGIS (using the tool value to points) per station and then used for model construction. An average map of salinity and SST covering the entire study period (1994–2011) was then used for model predictions (Fig. 2d,e).

### Model selection

Collinearity among explanatory variables may increase the probability of Type I errors; therefore, we tested for possible correlation between the environmental predictors. As survey data were both zero-inflated and over-dispersed, a two-stage approach was used to predict habitat suitability^53^. This two-step approach seemed suitable as a consequence of both sampling design and species behaviour, zero observations may indicate either low density (e.g. because of local extinctions caused by demographic stochasticity, or species not present at the time of survey) or true absence (e.g. habitat not suitable or species does not saturate its entire suitable habitat by chance^21^).

Generalised additive models (GAMs) were used to construct a two-part model consisting of a binomial (presence/absence) model (which predicts species occurrence) and a positive (truncated) abundance model (all zeroes excluded). The two models are combined by the multiplication of the predictions from both steps to obtain the final predicted value or preferential habitat model (also referred to as a delta model). The delta model was attempted for each species, but when its model evaluation was poor (see model evaluation section for details) only the results of the binomial occurrence model were presented.

GAMs are nonparametric regression techniques^54^ that allow for the modelling of relationships between variables without specifying any particular form for the underlying regression function. The use of smooth functions as regressors gives GAMs greater flexibility over linear (or other parametric) types of models. GAMs binomial occurrence models were developed using presence-absence data as the response variable and a logit link function (family binomial), to predict the mean presence probability of each of the species considered. GAMs positive models were developed using a subset containing only non-null densities and a log-link function (family negative binomial to account for overdispersion^53^), to predict the mean density on a log scale.

Starting from the full model, the most parsimonious model was selected on the basis of the lowest Akaike Information Criterion (AIC), corrected for small sample size (AICc). This approach selects the model with the best balance between bias and precision and avoids problems of, for example, multiple testing among explanatory variables^55^. A set of candidate models was compared using difference in AICc between the top-ranked and current model (delta AICc), and by calculating the AICc weight (the scaled likelihood that each model is the best description of the data^55^). Competing models of the best supported model were selected when having their AICc within 2 of the minimum^55^ and are presented in the Supplementary information (Tables S2–S3 online). Model goodness of fit was compared using the deviance and coefficient of determination (adj-r^2^). All modeling was carried out using the mgcv library in R v.3.0.2 software^56^,^57^.

### Model evaluation

Prior to model fitting, survey data were randomly divided into two datasets with 2/3 of the data used for model fitting (training dataset) and 1/3 for model evaluation (testing dataset). The two datasets were comparable as the environmental and abundance range of the evaluation dataset was within the overall range of the fitting dataset. Models were fitted on the training dataset and their performance was internally and externally (the latter using the testing dataset) evaluated. Both evaluations were conducted by comparing predictions in relation to the observations with Spearman’s rank correlation test (r_s_) corrected for spatial autocorrelation and implemented in SAM software^58^,^59^. Binomial models were tested for sensitivity by using the receiver operating characteristic (ROC) curve and assessed area under the receiver operating characteristic curve (AUC)^60^. An AUC value of 0.5 indicates that the model performs no better than a random model, whereas a value of 1 indicates that the model is capable of distinguising between occupied and unoccupied sites. AUC values of 0.7–0.9 indicate very good discrimination while values >0.9 indicate excellent. Finally, the predictive power of each model was assessed using a range of diagnostic plots^61^.

### Model Mapping

Maps of species predictions were constructed within the raster and rgdal libraries in R^62^ and then visualised in ArcGIS. The model error (defined as the absolute difference between observed and predicted species abundance) was also used to check and illustrate model fit. The spatial distribution of the model error was mapped by interpolation with ordinary kriging for each area^63^ and scaled between 0 to 1 (with a value of 1 corresponding to the maximum possible prediction error^23^).

## Results

### Environmental factors relevant to elasmobranch habitat selection

The five environmental variables were not collinear (Variance Inflation Factor <2^61^) and were tested for significant contribution to the models. Six delta models, including five single-species and one for community diversity, and four binomial habitat models were developed (Tables 3,4). Delta models were satisfactorily evaluated for *R. clavata*, *R. oxyrinchus*, *R. melitensis*, *S. blainvillei* and *D. licha* (Table 3) with species occurrence ranging between 5.95 and 20.30% over the total number of hauls (Table 1). Binomial models were developed and evaluated as satisfactory for four species: *C. monstrosa, M. mustelus, T. marmorata* and *C. granulosus* (Table 4) with species occurrence ranging between 8.10 and 22.60% over the total number of hauls (Table 1).

Depth was found to be the main environmental predictor in all nine species and community diversity habitat models (Tables 3 and 4) and in particular was the only factor influencing *C. monstrosa* habitat suitability (Table 4). Salinity and SST were important factors influencing the habitat suitability for all the species (found at least in one of the two models binomial or presence only). Slope was also a main predictor for community diversity and most of the species, with exception of *C. monstrosa* and *T. marmorata* (Tables 3 and 4). Finally, rugosity was found to affect elasmobranchs habitat suitability for the majority of species excluding *R. oxyrinchus*, *M. mustelus*, *C. monstrosa* and community diversity. Results of best models are summarized in Tables 3 and 4 while competing models are also presented in the Supplementary information (Tables S2–S3 online).

**Figure 3:**
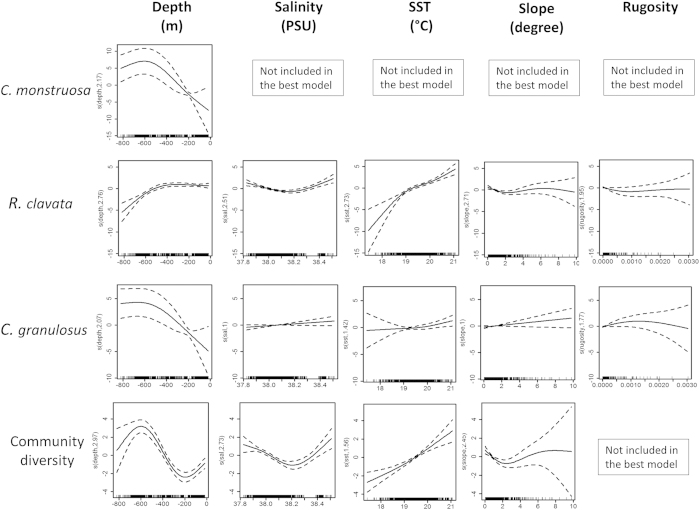
Partial GAM plots for the best binomial models for *C. monstrosa*, *R. clavata*, *C. granulosus* and Community diversity (number of species). Each plot represents the variable’s response shape, independent of the other variables, in relation to the probability of the species occurrence in the multivariate model. Salinity (PSU); SST: Sea Surface Temperature (°C); Slope (expressed in degrees) describes the rate of change in elevation over distance, its values range from 0 (flat terrain) to 90 degrees (steeper terrain); terrain rugosity captures variability in slope and aspect into a single measure, it ranges from 0 (no terrain variation) to 1 (complete terrain variation). The ranges of environmental variables are represented on the x-axis and the probability of the occurrence of the species is represented on the y-axis (logit scale). The degree of smoothing is indicated in the y-axis label. The dotted lines represent the 95% confidence intervals around the response curve.

Model behaviour showing species and community diversity-environment relationships is presented in Fig. 3 (with the examples of binomial models for *C. monstrosa*, *R. clavata*, *C. granulosus* and community diversity). The shape of the smoother of depth for *R. clavata* suggests that there is a negative nonlinear relationship associated with deeper waters for this species, with an inflexion between 400–500m depth (Fig. 3). In contrast both *C. monstrosa* and *C. granulosus* show a positive nonlinear trend with depth that suggests higher abundances in deeper waters. Nevertheless *C. monstrosa* shows an inflection at about 600 m depth, which indicates where the species reaches its optimum depth (Fig. 3). A different relationship with depth is shown for elasmobranchs community diversity. The shape of the smoother of depth has a peak at about 600 m, with an inflexion at about 200 m, suggesting higher diversity with deeper waters (>600 m; Fig. 3). The shape of the smoother for other environmental variables was quite similar for *R. clavata* and community diversity, whereby a positive relationship with SST and a negative curvilinear relationship with slope are shown in Fig. 3.

### Model evaluation

Model internal evaluation showed that both delta and binomial models performed well with strong positive correlations (corrected Spearman’s correlation test) between survey and predicted density values (Tables 3 and 4). All binomial models passed the sensitivity test suggesting that models had very good discriminating ability with AUC values ranging from a minimum of 0.78 (*T. marmorata*) to 0.94 (*C. monstrosa*). Finally, model external evaluation indicates that overall models performed well (significant positive correlation) when best models were tested on new data (testing dataset), with the exception of *T. marmorata* r_s_ = 0.07 and *D. licha* r_s_ = 0.09 which showed a weak correlation (Tables 3 and 4).

### Mapping model predictions and uncertainty

**Figure 4:**
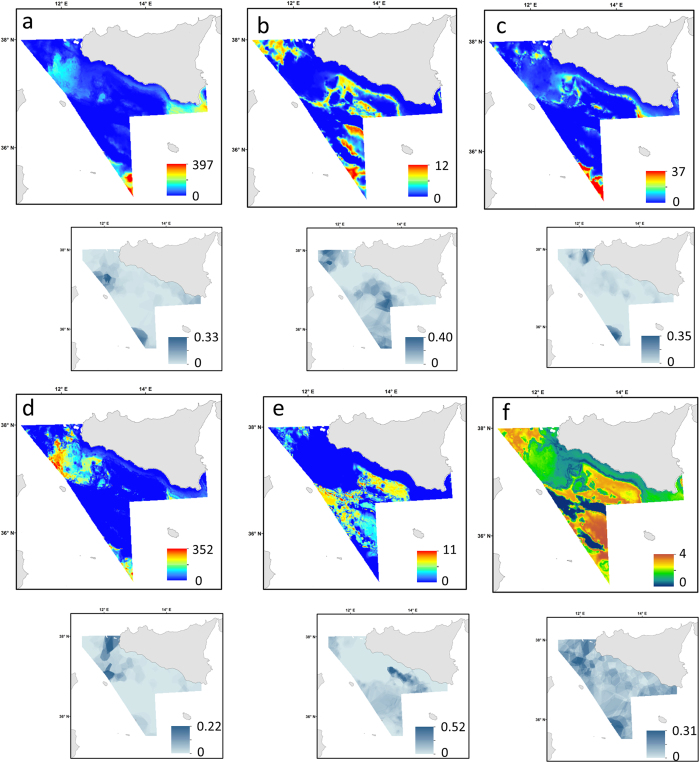
Predicted population densities (Nkm^−2^) and community diversity with delta models (main figure) representing preferential habitat and associated prediction error (small figure). (**a**) *Raja clavata,* (**b**) *Raja oxyrinchus*, (**c**) *Raja melitensis*, (**d**) *Squalus blainvillei*, (**e**) *Dalatias licha* (**f**) community diversity (37 species) in the Strait of Sicily. Prediction error maps: 0 and 1 correspond to the minimum and maximum possible errors, respectively. These maps were created with ArcGIS version 10.2.2 by Valentina Lauria.

**Figure 5:**
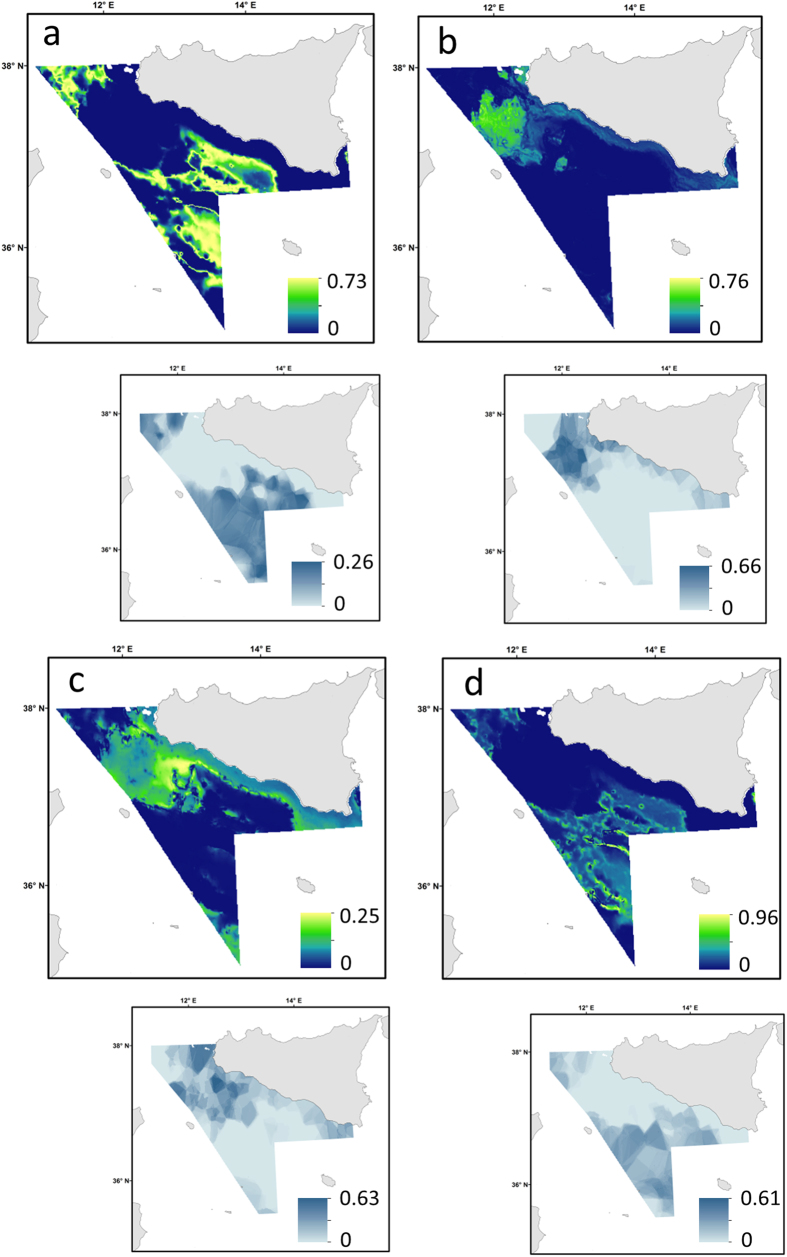
Predicted population densities (Nkm^−2^) with binomial models (main figure) representing preferential habitat and associated prediction error (small figure). (**a**) *Chimaera monstrosa*, (**b**) *Mustelus mustelus*, (**c**) *Torpedo marmorata* and (**d**) *Centrophorus granulosus* in the Strait of Sicily. Prediction error maps: 0 and 1 correspond to the minimum and maximum possible errors, respectively. These maps were created with ArcGIS version 10.2.2 by Valentina Lauria.

The maps of model predictions of all nine elasmobranch species and community diversity are presented in Figs 4 and 5. The predictive maps for the single-species models revealed that three different distribution patterns occurred across species as response of diverse habitat requirements. In particular, some species of ray and shark (e.g. *R. clavata*, *T. marmorata, M. mustelus, R. melitensis* and *S. blainvillei*) prefer shallow waters and coastal areas (Figs 4a,c,d and 5b,c), mainly corresponding to the Adventure bank and southern-east coast of Sicily (Fig. 1). This specific pattern relates to areas with lower values of slope (Fig. 2b) that are probably characterised by soft sediments. In contrast, *D. licha* and *C. granulosus* favour medium deep waters towards the central part of the Strait of Sicily (Figs 4e and 5d) that coincided with grounds of relatively higher values of slope, surface salinity and SST (Fig. 2). Finally, *R. oxyrinchus* and *C. monstrosa* (Figs 4b and 5a) show a preference for offshore areas with deeper waters and relatively high values of slope (Fig. 2b). The predictive habitat map for community diversity shows that deeper waters areas and part of the Adventure bank are associated with higher number of species than coastal waters (Fig. 4f). Model error maps for each habitat model revealed that, in general, higher model uncertainty corresponded to areas of higher predictions (zones where species were caught regularly; Figs 4 and 5).

## Discussion

Long-term fishery-independent survey data were used to improve our understanding of habitat selection by elasmobranchs in the central Mediterranean Sea in late spring-early summer. Our results and habitat maps revealed different habitat preferences among species and identified diversity hot-spots. Errors maps suggested that that species density/community diversity variability was greater in areas of higher densities.

Depth was found to be the main predictor in all nine elasmobranch habitat models suggesting species-specific relationships, while the effect of the other environmental factors was not consistent among species. In general, species showed three distribution patterns in relation to different depth stratum with the majority of species (e.g. *R. clavata*, *R. melitensis*, *T. marmorata, S. blainvillei* and *M. mustelus*) preferring the shallow waters of coastal areas and the Adventure bank (80–200 m depth). In contrast, the habitats of the two sharks *D. licha* and *C. granulosus* were associated with deeper waters (>600 m depth) of the southern part of the Strait of Sicily. Another distribution pattern was shown for one of the rays (*R. oxyrinchus*) and chimaera (*C. monstrosa*) that were found in medium depth waters (200–600 m depth). These patterns are probably related to different bathymetric conditions (e.g. light, food, temperature and currents) which can create *ad-hoc* habitat characteristics^64^. Our results for the Strait of Sicily confirm a bathymetric segregation previously observed for some species in this area^27^ and that is similar to other regions of the Mediterranean Sea^65^. For example, in the Gulf of Alicante (western Mediterranean), elasmobranchs are distributed in distinct areas in response to different depths with some minor overlapping between habitats^26^. Similarly, in the Aegean Sea (eastern Mediterranean) *M. mustelus* and *T. marmorata* are found in shallow areas (up to 180–200 m depth) of the continental shelf^28^,^66^, while *Raja asterias* prefers transitional depths between shelf and slope (about 200 m depth^28^).

Seafloor morphology (e.g. slope, rugosity) has been suggested to be an important factor that influences elasmobranch habitat suitability^52^,^67^,^68^,^69^. In the Strait of Sicily some species, such as *R. clavata*, *M. mustelus* and *S. blainvillei*, were associated with areas with minimum or gentle terrain variation (low slope and rugosity values which tend to be associated with fine sediment), while these factors did not seem to have an effect on the habitat preference of other species (e.g. *R. oxyrinchus* or *R. melitensis*). Species abundance was higher in shallow waters and the Adventure Bank, areas characterised by coarse calcareous sands (containing high proportions of bioclastic detritus) and silt^70^,^71^ which is possibly related to higher prey abundance (mainly small crustaceans and teleosts^72^). In general, our results agree with other studies where the elasmobranchs preference for sediment types varies amongst species and life stages^26^,^52^; however, some regional differences occurred. For example adults of *R. clavata* and *Mustelus* spp. seem to favour areas associated with coarse (e.g. gravel and pebbles) and sandy substrates in the eastern English Channel^52^ while in the Strait of Sicily these species seem to prefer mainly areas of deposition (silt and sand of the Adventure Bank, see Supplementary information Figure S1 online). Nevertheless we do not exclude the possibility that these distribution patterns might be marginally influenced by limitation in sampling data (as the MEDITS trawl survey reaches the maximum depth of 800 m and it is limited to soft substrata). This is because areas of deeper waters and hard substrata are generally difficult to trawl and may constitute a *de facto* refuge for certain species^73^. For example the spatial distribution of some elasmobranch species in the Celtic Sea (e.g. Dipturus spp.) has been suggested to be related to low commercial fishing effort and favourable habitat^73^, yet further research is required to understand how this *de facto* refuge influences elasmobranchs abundance and species richness.

SST, and to some degree salinity, were found to be predictors of elasmobranch habitat suitability in the Strait of Sicily in late spring-early summer. This is in agreement with other regional studies (eastern English Channel, western Mediterranean Sea^26^,^52^) which suggested that these environmental factors are relevant to elasmobranch habitat selection. Our results show a positive curvilinear relationship between species abundance and SST (*R. clavata* and community diversity Fig. 3); in addition the prediction maps suggest greater species density (e.g. *T. marmorata*) in areas where SST is higher (circa 21 °C).

SST is strongly related with primary productivity and previous studies on elasmobranchs have suggested that SST is an important factor that regulates their ecology and habitat selection. The majority of elasmobranchs are ectothermic and changes in the environmental temperature affect most physiological processes^74^,^75^. In addition SST indirectly influences elasmobranchs distribution and movement patterns through availability of preferred prey which make any one area more suitable than another^76^. For example Pennino *et al.* (2013)^26^ found that the abundance of a shark (*S. canicula*) in the western Mediterranean was higher in areas with low productivity and SST usually associated with deeper waters. The effect of salinity was not clear on elasmobranch distribution (Fig. 3), probably due to the fact that the study area is characterised by a very limited gradient with salinity ranging between 37.9–38.3 PSU (Fig. 2d); for this reason it is not likely to be a major influence in the study area.

Our predictive map suggests that community diversity (number of species) is higher in deeper waters (with a peak at about 600 m depth) in late spring-early summer. These areas coincided with grounds of relatively higher values of slope, surface salinity and SST; however some parts of the Adventure Bank (about 100 m depth) were also predicted as elasmobranchs diversity hot-spots (Fig. 4f). This result is in agreement with other studies that showed regional differences in the optimum distribution of elasmobranch community, for example while in the eastern Mediterranean (Aegean Sea) the elasmobranchs community can be found up to 700 m depth with a peak about 180–430 m depth^77^, in the western part (Balearic Islands) the optimum of species distribution occurs at about 200–300 m depth^43^.

Conservation of rare and endangered species can be informed using predictive distribution modelling^78^, even if the application of these tools can be challenging as survey data are often zero-inflated and overdispersed^79^. In this study, delta models were able to predict the probability of species occurrence in the central Mediterranean Sea, although in some cases binomial models performed better and were preferred to delta models (based on the internal and external evaluation; Tables 3 and 4). This is similar to Martin *et al.*, (2012)^52^ who suggested that the frequency of occurrence of species in fishery survey data can affect the performance of habitat models. This is because positive models are constructed on the positive observations (species relative abundance > 0) of the data, which account for a smaller portion of the original dataset, and can influence the model performance in terms of evaluation and calibration^52^. In contrast, within the binomial models even zeroes provide information (i.e. the species was not captured at these locations because the habitat is not suitable) which could result in better prediction maps. The advantage of using delta models is that the model uses all the data available to predict species distribution. In our study, delta models were successfully applied to species with an overall occurrence in the original data higher than 7% (e.g. *R. clavata*, *R. oxyrinchus*, *R melitensis* and *S. blainvillei*; Table 2). The other species preferential habitats were modelled using binomial models (see methods section; Table 4). This suggests that delta models are a valid tool to model rare species (when data are zero-inflated and overdispersed^22^,^79^) but it is difficult to apply a clear cut-off (based only on the occurrence of a specific species in the dataset) on which model (delta or binomial only) to use for any given case.

## Conclusion

This study enhances our understanding of habitat preference and hot-spot distribution for the conservation of elasmobranchs in the central Mediterranean Sea (Figs 4 and 5). Environmental factors, in particular depth, seafloor morphology and SST, were the main drivers for elasmobranch habitat suitability in late spring-early summer. Identifying and protecting critical habitats (for threatened species and community diversity) is one of the main uses of habitat suitability models for conservation purpose^25^. Seasonal patterns in the relative abundance of elasmobranchs are generally observed as a response to reproduction, migration and foraging^75^,^80^. While fairly stables distribution patterns have been suggested across seasons (Autumn and Summer) for some species (e.g. *R. clavata*, *Raya montagui* and *S. canicula*)^81^ in the Northeast Atlantic this may be different in the Mediterranean Sea. Data on seasonal differences in the distributions of elasmobranchs in the Strait of Sicily are limited. Ragonese *et al.*, (2013)^27^ showed that some chimaera and sharks species (e.g. *C. monstrosa*, *M. mustelus, C. granulosus*) exhibit slightly different patterns across seasons (autumn and summer) with highest abundance in summer. Similarly in other parts of the Mediterranean Sea (i.e. Aegean Sea) Damals *et al.*, (2009)^80^ showed that the distribution patterns of some rays (*Leucoraja naevus*) was favoured during spring and summer than winter months (the abundance increases favouring warm waters) probably as function of recruitment. Conversely, *R. clavata* shows a stable distribution pattern across seasons (probably due to a very narrow temperature ranges) but increases its abundance during summer. Although the present study was limited only to the late spring-early summer period and it does not capture the fully extent of distributional patterns of elasmobranchs, it still highlights the habitat utilisation of sensitive and data deficient species in the central Mediterranean Sea. For these reasons similar studies on the distribution of elasmobranchs covering other times of the year (seasons) are necessary to support conservation plans in this area. Following the International Plan of Action for the Conservation and Management of Sharks (1999), the Action Plan for the Conservation of Cartilaginous Fishes in the Mediterranean (2003) and the EU Action Plan for the Conservation and Management of Sharks (2009), the General Fisheries Commission for the Mediterranean (GFCM) organised a working group with the aim of increase the knowledge on the biology (e.g. distribution, population dynamics) and fishery of elasmobranchs in many parts of the Mediterranean Sea^82^. The predictive habitat maps produced in this study (Figs 4 and 5) represent the first attempt to identify key areas and distribution patterns for habitat protection of elasmobranch species and community diversity in the central Mediterranean Sea. In the Strait of Sicily most of the ray and shark species are sold at fish markets, while species that have no commercial value are discarded (Table 2). The impact of the fishery has partially decreased in this area over time^83^; however, the implementation of lasting and effective conservation and fishery management plans, following the Marine Strategy Framework Directive^30^, are still lacking. An improved knowledge of elasmobranch habitat suitability is therefore necessary to effectively manage commercial stocks, but also to carefully protect species of conservation interest^15^,^84^. The results of this study, including the predictive map of elasmobranch community diversity (Fig. 4f), will inform GFCM’s future conservation programs^85^ as well as provide a base for the design of fishery-oriented MPA networks in the Mediterranean Sea.

This study is in line with the objectives of the European Commission within the framework of an ecosystem-based approach for fisheries management, which aims to identify priority conservation areas to maintain sustainable marine living resources^86^. It provides a base for future research on elasmobranchs in the central Mediterranean in order to prevent the extinction of species before we understand their full importance in the marine ecosystem.

## Additional Information

**How to cite this article**: Lauria, V. *et al.* Predictive habitat suitability models to aid conservation of elasmobranch diversity in the central Mediterranean Sea. *Sci. Rep.* **5**, 13245; doi: 10.1038/srep13245 (2015).

## Supplementary Material

Supplementary material

## Figures and Tables

**Table 1:** Elasmobranch species captured during the MEDITS Survey (1994–2011) and for which community diversity index is calculated.

Latin name	Species authorship	Family	Common name	Conservation status IUCN	Occurrence%
ORDER CARCHARHINIFORMES					
* Galeus melastomus*	Rafinesque, 1810	Scyliorhinidae	Black mouth catshark	Least concern	43.57
* Mustelus asterias*	Cloquet, 1821	Triakidae	Starry Smoothhound	Least concern	0.52
* Mustelus punctulatus*	Risso, 1826	Triakidae	BlackspottedSmoothhound	Data deficient	0.74
* Mustelus mustelus*	Linnaeus, 1758	Triakidae	Common Smoothhound	Vulnerable	8.55
* Scyliorhinus canicula*	Linnaeus, 1758	Scyliorhinidae	Lesser Spotted Dogfish	Least concern	29.89
* Scyliorhinus stellaris*	Linnaeus, 1758	Scyliorhinidae	Nursehound	Near Threatened	0.45
* Chimaera monstrosa*	Linnaeus, 1758	Chimaeridae	Rabbit fish	Near Threatened	22.60
ORDER HEXANCHIFORMES					
* Heptranchias perlo*	Bonnaterre, 1788	Hexanchidae	One-finned Shark	Near Threatened	2.01
* Hexanchus griseus*	Bonnaterre, 1788	Hexanchidae	Bluntnose Sixgill Shark	Near Threatened	0.37
ORDER RAJIFORMES					
* Dasyatis pastinaca*	Linnaeus, 1758	Dasyatidae	Common stingray	Data deficient	1.34
* Myliobatis aquila*	Linnaeus, 1758	Myliobatidae	Common Eagle Ray	Data deficient	0.37
* Pteromylaeus bovinus*	Geoffroy Saint-Hilaire, 1817	Myliobatidae	Bullray	Data deficient	0.22
* Raja alba*	Lacepède, 1803	Rajidae	Bottlenose Skate	Endangered	0.89
* Raja asterias*	Delaroche, 1809	Rajidae	Starry ray	Least concern	4.68
* Dipturus batis*	Linnaeus, 1758	Rajidae	Blue Skate	Critically endangered	0.07
* Raja brachyuran*	Lafont, 1873	Rajidae	Blonde ray	Near Threatened	0.45
* Raja circularis*	Couch, 1838	Rajidae	Sandy ray	Vulnerable	0.97
* Raja clavata*	Linnaeus, 1758	Rajidae	Thornback Skate	Near Threatened	20.30
* Raja fullonica*	Linnaeus, 1758	Rajidae	Shagreen Ray	Near Threatened	0.07
* Raja melitensis*	Clark, 1926	Rajidae	Maltese skate	Critically endangered	7.29
* Raja miraletus*	Linnaeus, 1758	Rajidae	Brown skate	Least concern	18.88
* Raja montagui*	Fowler, 1910	Rajidae	Spotted ray	Least concern	6.25
* Raja naevus*	Müller & Henle, 1841	Rajidae	Cuckoo Ray	Least concern	0.15
* Raja oxyrinchus*	Linnaeus, 1758	Rajidae	Long-nosed Skate	Near Threatened	10.86
* Raja polystigma*	Regan, 1923	Rajidae	Speckled Ray	Near Threatened	0.22
* Raja radula*	Delaroche, 1809	Rajidae	Rough Ray	Data deficient	0.15
* Torpedo marmorata*	Risso, 1810	Torpedinidae	Marbled electric ray	Data deficient	8.25
* Torpedo nobiliana*	Bonaparte, 1835	Torpedinidae	Great Torpedo Ray	Data deficient	1.93
* Torpedo torpedo*	Linnaeus, 1758	Torpedinidae	Common torpedo	Data deficient	0.52
ORDER SQUALIFORMES					
* Centrophorus granulosus*	Bloch & Schneider, 1801	Centrophoridae	Gulper shark	Vulnerable	8.10
* Centrophorus uyato*	Rafinesque, 1810	Centrophoridae	Little gulper shark	Data deficient	0.52
* Etmopterus spinax*	Linnaeus, 1758	Etmopteridae	Velvet belly lanternshark	Least concern	29.37
* Oxynotus centrina*	Linnaeus, 1758	Oxynotidae	Angular Rough Shark	Vulnerable	0.74
* Dalatias licha*	Bonnaterre, 1788	Dalatiidae	Kitefin shark	Near Threatened	5.95
* Squalus acanthias*	Linnaeus, 1758	Squalidae	Spurdog	Vulnerable	0.30
* Squalus blainvillei*	Risso, 1827	Squalidae	Longnosespurdog	Data deficient	13.16

Occurrence describes the number of hauls in which the species was found (expressed as percentage).

**Table 2:** Details of survey data utilised in this study.

Scientific name	Concern IUCN	Fishery	Model development and internal evaluation		Model external evaluation	
			Total hauls	Occurrence %	Total hauls	Occurrence %
CHIMAERA						
*Chimaera monstrosa*	Near Threatened	Totally discarded (Ragonese *et al.* 2013)	927	22.11	418	23.68
RAYS						
*Raja clavata*	Near Threatened	Sold at the market (Serena, 2014)	927	20.39	418	20.10
*Raja oxyrinchus*	Near Threatened	Sold at the market (Tudela, 2004)	927	10.90	418	10.77
*Raja melitensis*	Critically endangered	Totally discarded (Damalas and Vassilopoulou, 2009)	927	7.12	418	7.66
*Torpedo marmorata*	Data deficient	Sold at the market (Giusto B. pers comments)	927	7.77	418	9.33
SHARKS						
*Mustelus mustelus*	Vulnerable	Sold at the market (Ragonese *et al.* 2013)	927	9.06	418	7.42
*Squalus blainvillei*	Data deficient	Sold at the market (Ragonese *et al.* 2013)	927	13.92	418	11.48
*Dalatias licha*	Near Threatened	Mainly discarded (Arena, 1985)	927	6.15	418	5.50
*Centrophorus granulosus*	Vulnerable	Mainly discarded (Ragonese *et al.* 2013)	927	8.09	418	8.13

Hauls is the number of trawl hauls used for model development and evaluation, this was associated with a full set of environmental variables. Occurrence describes the number of hauls in which the species was found (expressed as percentage).

**Table 3:**
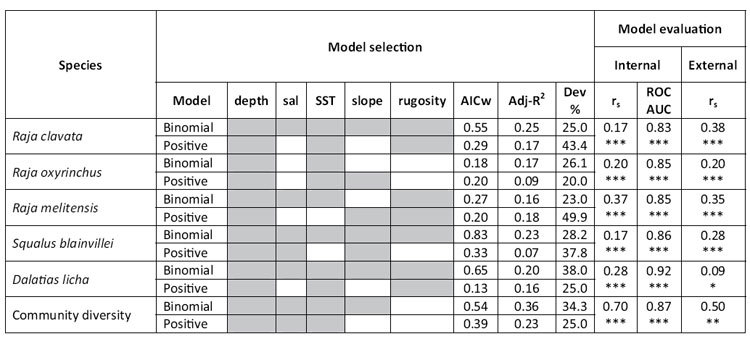
Selected models for five demersal elasmobranchs and Community diversity (species richness index constructed on 37 species) using delta models.

Predictors include depth, surface salinity (sal), sea surface temperature (SST), slope and rugosity. Only the best supported models are shown (variables included in model are shaded in grey); AICc weight: Akaike’s Information Criteria (corrected) weights, values range from 0 to 1, and high values indicate strong support for a given predictor. Models were evaluated by R^2^-adjusted coefficient and deviance (Dev): percentage of deviance explained. Only for the binomial model the Receiver Operating Characteristic (ROC) and Area Under the Curve (AUC) were calculated. Significance value of the Spearman’s correlation coefficient (r_s_) (corrected for spatial autocorrelation) for the delta model is given as ***p value < 0.001, **p value < 0.01, *p value < 0.05.

**Table 4:**
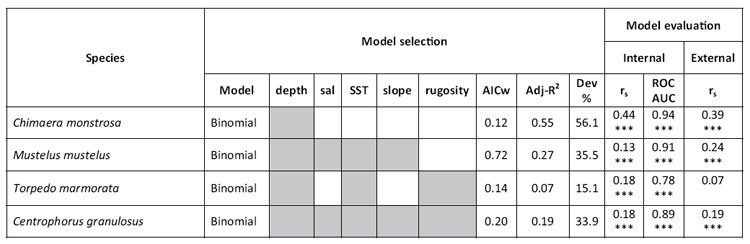
Selected models for four demersal elasmobranchs using binomial models.

Predictors include depth, surface salinity (sal), sea surface temperature (SST), slope and rugosity. Only the best supported models are shown (variables included in model are shaded in grey); AICc weight: Akaike’s Information Criteria (corrected) weights, values range from 0 to 1, and high values indicate strong support for a given predictor. Models were evaluated by R^2^-adjusted coefficient and deviance (Dev): percentage of deviance explained. Only for the binomial model the Receiver Operating Characteristic (ROC) and Area Under the Curve (AUC) were calculated. Significance value of the Spearman’s correlation coefficient (r_s_) (corrected for spatial autocorrelation) for the delta model is given as ***p value < 0.001, **p value <0.01, *p value < 0.05.
