# Supplementary material for: Predictive habitat suitability models to aid conservation of elasmobranch diversity in the central Mediterranean Sea
Source: Sci Rep. 2015 Aug 14;5:13245. doi: 10.1038/srep13245 (PMC4536484; doi:10.1038/srep13245)

Supplementary material

Article: Predictive habitat suitability models to aid conservation of elasmobranchs diversity in the central Mediterranean Sea

V. Lauria, M. Gristina, M.J. Attrill, F. Fiorentino & G. Garofalo

Table S1. Dates of the MEDITS survey in the Strait of Sicily from 1994 to 2011.

CRUISE	Vessel	Start	End
MEDITS	S. Anna	11-Jun-94	15-Jul-94
MEDITS	S. Anna	03-Jun-95	17-Jun-95
MEDITS	S. Anna	31-May-96	12-Jun-96
MEDITS	S. Anna	03-Jun-97	12-Jun-97
MEDITS	S. Anna	16-Jun-98	27-Jun-98
MEDITS	S. Anna	28-May-99	09-Jun-99
MEDITS	S. Anna	26-May-00	08-Jun-00
MEDITS	S. Anna	19-May-01	01-Jun-01
MEDITS	S. Anna	11-Jul-02	24-Aug-02
MEDITS	S. Anna	13-Jul-03	13-Aug-03
MEDITS	S. Anna	10-Jun-04	11-Jul-04
MEDITS	S. Anna	05-Jul-05	13-Aug-05
MEDITS	S. Anna	19-May-06	14-Jun-06
MEDITS	S. Anna	10-Jun-07	09-Jul-07
MEDITS	S. Anna	12-May-08	06-Jun-08
MEDITS	S. Anna	21-May-09	16-Jun-09
MEDITS	S. Anna	20-May-10	24-Jun-10
MEDITS	S. Anna	24-Jun-11	02-Aug-11

Table S2. Competing habitat suitability models for demersal elasmobranchs and community diversity using the delta model. Predictors include depth, surface salinity (sal), sea surface temperature (SST), slope and rugosity (variables included in model are shaded in grey); AICc weight: Akaike's Information Criteria (corrected) weights, values range from 0 to 1, and high values indicate strong support for a given predictor. Models were evaluated by R^2 adjusted coefficient and deviance (Dev): percentage of deviance explained.

Species	Model selection								
	Model	depth	sal	SST	slope	rugosity	AICw	Adj-R^2	Dev %
Raja clavata	Binomial	■	■	■	■		0.39	0.24	24.5
	Positive	■		■	■	■	0.22	0.17	44.7
	Positive	■	■	■		■	0.13	0.17	43.5
Raja oxyrinchus	Binomial	■	■	■			0.17	0.17	26.5
	Binomial	■		■		■	0.16	0.18	26.3
	Binomial	■	■	■		■	0.15	0.18	26.8
	Binomial	■		■	■		0.11	0.18	26.2
	Binomial	■	■	■	■		0.09	0.18	26.6
	Positive	■			■		0.12	0.10	18.0
	Positive	■					0.10	0.08	15.9
	Positive	■		■			0.10	0.08	17.0
Raja melitensis	Binomial	■	■	■	■		0.23	0.12	22.9
	Binomial	■	■				0.18	0.14	22.1
	Binomial	■	■	■			0.11	0.15	21.5
	Positive	■				■	0.18	0.20	47.9
	Positive	■	■		■	■	0.12	0.18	51.0
	Positive	■	■	■	■	■	0.12	0.18	53.8
	Positive	■	■			■	0.10	0.20	49.0
	Positive	■		■	■	■	0.09	0.16	50.9
	Positive	■	■	■		■	0.08	0.15	50.9
Squalus blainvillei	Binomial	■	■	■	■		0.30	0.07	38.5
Dalatias licha	Positive	■	■	■		■	0.12	0.20	28.5
	Positive	■		■		■	0.11	0.16	25.0
	Positive	■	■	■			0.07	0.17	23.7
Centrophorus granulosus	Binomial	■	■	■		■	0.16	0.19	33.4
	Binomial	■		■		■	0.14	0.19	33.0
	Binomial	■		■	■	■	0.14	0.19	33.3
	Binomial	■	■	■	■		0.11	0.18	32.9
	Binomial	■		■	■		0.08	0.17	32.4
Community diversity	Binomial	■	■	■	■	■	0.46	0.36	34.5
	Positive	■		■			0.22	0.23	24.0
	Positive	■	■	■	■		0.17	0.24	25.1

Table S3. Competing habitat suitability models for demersal elasmobranchs using the binomial model. Predictors include depth, surface salinity (sal), sea surface temperature (SST), slope and rugosity (variables included in model are shaded in grey); AICc weight: Akaike's Information Criteria (corrected) weights, values range from 0 to 1, and high values indicate strong support for a given predictor. Models were evaluated by R^2 adjusted coefficient and deviance (Dev): percentage of deviance explained.

<table>
<tr><th rowspan="2">Species</th><th colspan="9">Model selection</th></tr>
<tr><th>Model</th><th>depth</th><th>sal</th><th>SST</th><th>slope</th><th>rugosity</th><th>AICw</th><th>Adj-R^2</th><th>Dev %</th></tr>
<tr><td rowspan="8">Chimaera monstrosa</td><td>Binomial</td><td bgcolor="#c0c0c0"></td><td bgcolor="#c0c0c0"></td><td></td><td></td><td bgcolor="#c0c0c0"></td><td>0.11</td><td>0.55</td><td>56.6</td></tr>
<tr><td>Binomial</td><td bgcolor="#c0c0c0"></td><td bgcolor="#c0c0c0"></td><td></td><td></td><td></td><td>0.11</td><td>0.55</td><td>54.4</td></tr>
<tr><td>Binomial</td><td bgcolor="#c0c0c0"></td><td></td><td bgcolor="#c0c0c0"></td><td></td><td bgcolor="#c0c0c0"></td><td>0.09</td><td>0.55</td><td>56.5</td></tr>
<tr><td>Binomial</td><td bgcolor="#c0c0c0"></td><td></td><td></td><td></td><td bgcolor="#c0c0c0"></td><td>0.09</td><td>0.55</td><td>56.2</td></tr>
<tr><td>Binomial</td><td bgcolor="#c0c0c0"></td><td></td><td bgcolor="#c0c0c0"></td><td></td><td></td><td>0.08</td><td>0.55</td><td>56.2</td></tr>
<tr><td>Binomial</td><td bgcolor="#c0c0c0"></td><td bgcolor="#c0c0c0"></td><td bgcolor="#c0c0c0"></td><td></td><td bgcolor="#c0c0c0"></td><td>0.06</td><td>0.55</td><td>56.6</td></tr>
<tr><td>Binomial</td><td bgcolor="#c0c0c0"></td><td bgcolor="#c0c0c0"></td><td bgcolor="#c0c0c0"></td><td></td><td></td><td>0.05</td><td>0.55</td><td>56.3</td></tr>
<tr><td>Binomial</td><td bgcolor="#c0c0c0"></td><td bgcolor="#c0c0c0"></td><td></td><td bgcolor="#c0c0c0"></td><td bgcolor="#c0c0c0"></td><td>0.04</td><td>0.55</td><td>56.6</td></tr>
<tr><td>Mustelus mustelus</td><td>Binomial</td><td bgcolor="#c0c0c0"></td><td bgcolor="#c0c0c0"></td><td bgcolor="#c0c0c0"></td><td bgcolor="#c0c0c0"></td><td bgcolor="#c0c0c0"></td><td>0.28</td><td>0.27</td><td>35.5</td></tr>
<tr><td rowspan="6">Torpedo marmorata</td><td>Binomial</td><td bgcolor="#c0c0c0"></td><td></td><td bgcolor="#c0c0c0"></td><td bgcolor="#c0c0c0"></td><td></td><td>0.13</td><td>0.08</td><td>15.1</td></tr>
<tr><td>Binomial</td><td bgcolor="#c0c0c0"></td><td></td><td bgcolor="#c0c0c0"></td><td bgcolor="#c0c0c0"></td><td bgcolor="#c0c0c0"></td><td>0.12</td><td>0.07</td><td>15.4</td></tr>
<tr><td>Binomial</td><td bgcolor="#c0c0c0"></td><td></td><td></td><td></td><td bgcolor="#c0c0c0"></td><td>0.11</td><td>0.07</td><td>14.5</td></tr>
<tr><td>Binomial</td><td bgcolor="#c0c0c0"></td><td></td><td></td><td bgcolor="#c0c0c0"></td><td bgcolor="#c0c0c0"></td><td>0.08</td><td>0.07</td><td>14.8</td></tr>
<tr><td>Binomial</td><td bgcolor="#c0c0c0"></td><td></td><td></td><td bgcolor="#c0c0c0"></td><td></td><td>0.07</td><td>0.07</td><td>14.4</td></tr>
<tr><td>Binomial</td><td bgcolor="#c0c0c0"></td><td bgcolor="#c0c0c0"></td><td bgcolor="#c0c0c0"></td><td></td><td bgcolor="#c0c0c0"></td><td>0.06</td><td>0.07</td><td>15.2</td></tr>
<tr><td rowspan="5">Centrophorus granulosus</td><td>Binomial</td><td bgcolor="#c0c0c0"></td><td bgcolor="#c0c0c0"></td><td bgcolor="#c0c0c0"></td><td></td><td bgcolor="#c0c0c0"></td><td>0.16</td><td>0.19</td><td>33.4</td></tr>
<tr><td>Binomial</td><td bgcolor="#c0c0c0"></td><td></td><td bgcolor="#c0c0c0"></td><td></td><td bgcolor="#c0c0c0"></td><td>0.14</td><td>0.19</td><td>33.0</td></tr>
<tr><td>Binomial</td><td bgcolor="#c0c0c0"></td><td></td><td bgcolor="#c0c0c0"></td><td bgcolor="#c0c0c0"></td><td bgcolor="#c0c0c0"></td><td>0.14</td><td>0.19</td><td>33.3</td></tr>
<tr><td>Binomial</td><td bgcolor="#c0c0c0"></td><td bgcolor="#c0c0c0"></td><td bgcolor="#c0c0c0"></td><td bgcolor="#c0c0c0"></td><td></td><td>0.11</td><td>0.18</td><td>32.9</td></tr>
<tr><td>Binomial</td><td bgcolor="#c0c0c0"></td><td></td><td bgcolor="#c0c0c0"></td><td bgcolor="#c0c0c0"></td><td bgcolor="#c0c0c0"></td><td>0.08</td><td>0.17</td><td>32.4</td></tr>
</table>

Figure S1. Sediment distribution in the Adventure Bank (adapted from Colantoni, P. Carta Batimetrica-Morfologica-Litologica del Banco Avventura (Canale di Sicilia). Consiglio Nazionale delle Ricerche Progetto Finalizzato Oceanografia e Fondi Marini. Istituto Geografico De Agostini, Novara, 1988). The original map was georeferenced with ArcGIS ArcView EDU PRO 9.3.1 by Germana Garofalo.

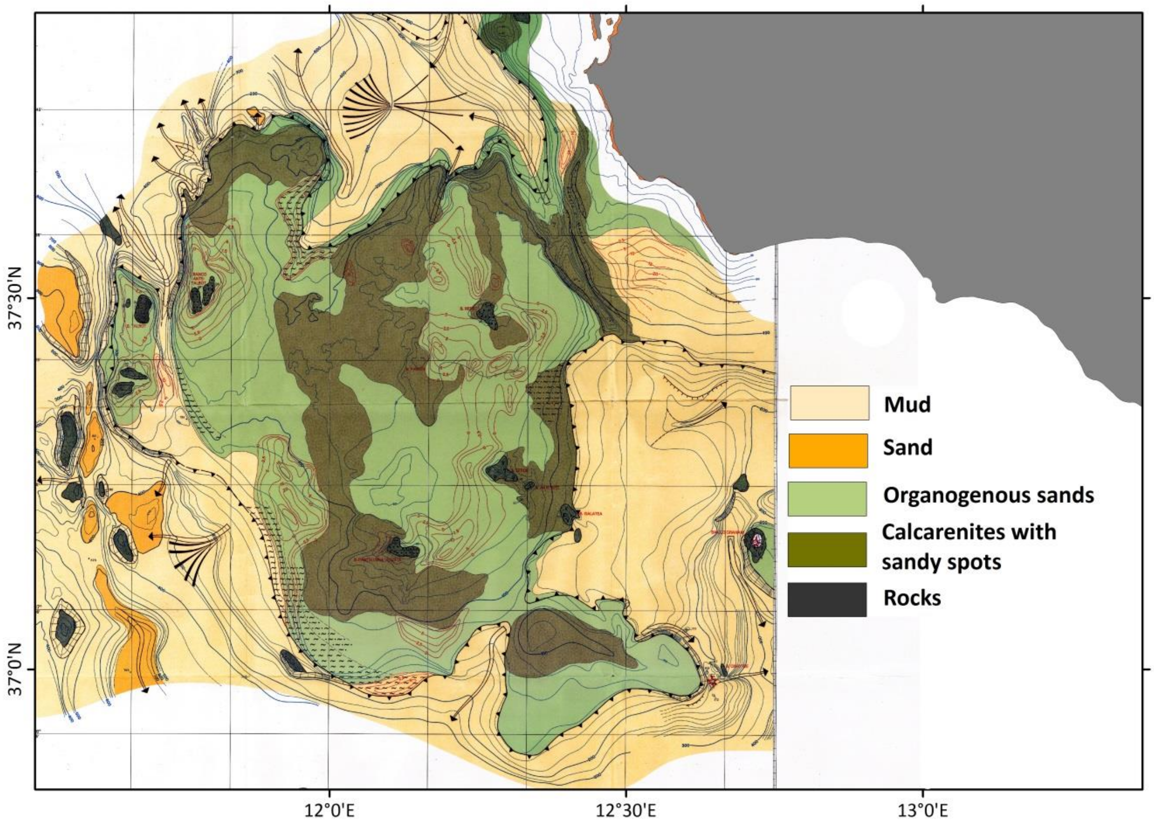

Supplement: Supplementary material [file srep13245-s1.pdf]
